# Time and tide: Seasonal, diel and tidal rhythms in Wadden Sea Harbour porpoises (*Phocoena phocoena*)

**DOI:** 10.1371/journal.pone.0213348

**Published:** 2019-03-20

**Authors:** Beate Zein, Benno Woelfing, Michael Dähne, Tobias Schaffeld, Stefan Ludwig, Jacob Hansen Rye, Johannes Baltzer, Andreas Ruser, Ursula Siebert

**Affiliations:** 1 Institute for Terrestrial and Aquatic Wildlife Research, University of Veterinary Medicine Hannover, Foundation, Büsum, Germany; 2 School of Geography & Geosciences, University of St Andrews, St Andrews, Fife, Scotland, United Kingdom; 3 German Oceanographic Museum, Stralsund, Mecklenburg-Vorpommern, Germany; 4 German Navy Headquarters, Geo-Information Division, METOC Support, Rostock, Germany; 5 The Danish Coastal Authority, Ministry of Environment and Food of Denmark, Lemvig, Denmark; Sanya Institute of Deep-sea Science and Engineering Chinese Academy of Sciences, CHINA

## Abstract

Odontocetes have evolved a rich diversity of prey- and habitat-specific foraging strategies, which allows them to feed opportunistically on locally and temporally abundant prey. While habitat-specific foraging strategies have been documented for some odontocete species, this is less known for the harbour porpoise (*Phocoena phocoena*). We collected multiple years of acoustic data using echolocation click loggers to analyse porpoise occurrence and buzzing behaviour, indicating feeding, in the German Wadden Sea (North Sea). Seasonal, diel and tidal effects were studied using Generalised Estimating Equations (GEE-GAMs). Locally season, time of day and tidal time significantly influenced the probability of porpoise detections and detection of foraging sequences (buzzes). Hunting strategies, and therefore frequency of buzzes, were likely affected by prey distribution and large differences between POD locations indicated that porpoises used highly specific behaviour adapted to tide and time of day to efficiently feed on the available prey. Strong seasonal and spatial variation in diel and tidal effects underline the importance of long-term observations. Studies on porpoise behaviour are often based on short-term observations and might rather reflect a seasonal than a general pattern. The results of this study show clearly that significant changes in porpoise behaviour can be found in short and long-term observations. Here some features are based on short term determinants and others are stable over years and care should be taken about drawing general conclusions based on local patterns. Highly variable spatio-temporal patterns indicate a high flexibility of porpoises in a highly variable environment and address a challenge for complex conservation management plans.

## Introduction

In a fast-changing environment with variable prey abundance, odontocetes have evolved highly adaptive foraging strategies. For an optimal foraging success, predators need to locate prey, which might be in localised patches due to biotic or abiotic factors [1,2]. While habitat-specific foraging strategies have been documented for some odontocete species, this is less known for the harbour porpoise (*Phocoena phocoena*). The dependency of predators’ distribution to prey availability should be especially high for harbour porpoises because they have a high energy demand due to their small size and their life in temperate waters [3–5].

In the North-East Atlantic, coastal and shelf waters are common habitats of the harbour porpoise [6]. In the North Sea it is the most common marine mammal [6] occurring both in offshore areas in the central, western and northern parts, and in coastal waters in the Wadden Sea in southern parts. There are major differences between those two habitats. The offshore areas of the North Sea are 30 to 200 metres deep and the Wadden Sea is mostly shallower than 20 metres. This affects the water temperatures throughout the year resulting in warmer waters in the Wadden Sea in spring and summer [7,8]. Other abiotic factors strongly affecting shallow areas of the North Sea are solar radiation, wind and tide [9]. Additionally, in coastal zones the effects of rivers, estuaries and tidal flats (Wadden Sea) lead to an increase in the concentration of organic matter [10] and hence might increase the prey availability, which is linked to porpoise distribution [11–13].

Porpoise occurrence varies seasonally [14,15] and gradually over longer timescales [16] in the North Sea and indicate complex and variable pattern of porpoise occurrence. High porpoise occurrences have been detected in winter, spring and summer in different inshore areas in various countries [14,16–27]. In offshore areas in Germany a peak of porpoise occurrence was found in autumn and winter [28] which might suggest a seasonal movement of harbour porpoises between inshore and offshore areas [29,30]. In general, coastal habitats are predominantly used in summer time in the North Sea [22,25] and a widespread distribution with a shift towards offshore areas is found in autumn [14,19,29–31].

On a smaller scale, local porpoise abundance can change with changing abiotic factors such as fronts, day length, water depth, distance to coast and residual currents [15,30]. The occurrence of porpoises has been found to be influenced by currents [15,32] and tidally driven in- and outflows [33–35]. Tides generally have an effect on the distribution of porpoises [36–39] but the impact is highly specific to sites and tidal states [33,40]. Depending on the site monitored, animal detection probability has been found to peak at high tide [26,41–43], but also flood tide [36,41,44], ebb tide [45], slack water [26] or with no tidal effects [46]. Another abiotic factor that has been found to influence porpoise occurrence are diel phases. Variable effects were found using acoustic methods [17,28,40,47]. Slight tendencies towards nocturnal detections and feeding behaviour are most common [26,47–52] as well as for dusk and dawn [35,43,53].

Porpoises are traditionally monitored using visual surveys in large areas at large intervals [6,54] or in smaller areas at shorter intervals [14,15]. These visual monitoring methods have scale dependent advantages–large scale surveys may be the best solution to monitor the population, while shorter intervals will lead to improved power to detect short-term changes in occurrence. While porpoises can be observed visually at the surface at good sea conditions and during day light hours only, other methods are needed to detect porpoises more continuously. Therefore, we can detect echolocation signals used by toothed whales for navigation [55,56] and to find prey [57–59]. The emitted high frequency clicks can be used to detect porpoises in monitoring studies [19,28,49,60]. Apart from occurrence, click sequences can give insight into porpoise behaviour [58,59,61]. Recordings of click sequences with very short inter-click intervals (< 10 milliseconds) can serve as a proxy to categorize foraging behaviour in acoustic data [47,49,59]. A method that can detect porpoises continuously with very limited spatial coverage, but high temporal resolution is stationary acoustic monitoring (SAM) [17,60]. It can collect data on 24-hour cycles and therefore allows for analysing diel cycles since it does not depend on daylight [52]. Therefore, it is a useful method to study short term and long term changes of porpoise behaviour.

Knowledge on movements of porpoises in the North Sea is still limited and large-scale, long-term studies are necessary to unravel the distribution in time and space over annual cycles. In our study, we analysed a long-term passive acoustic data set to investigate harbour porpoise occurrences and feeding behaviour in the German Wadden Sea. We tested if (1) season, day time and/or tide drive changes in porpoise occurrence in the Wadden Sea and (2) if buzzing behaviour, as a proxy for feeding is influenced by the same factors.

## Methods

### Ethical statement

Permissions for the deployment of measurement positions were given from the Federal Waterways and Shipping Administration (WSV) and Agency for Coastal Defence, National Park and Marine Conservation Schleswig- Holstein and Lower Saxony. No other specific permissions were required for these locations and activities. Also, no ethical approval is required because no handlings of animals or experiments on animals were conducted and we confirm that the field studies did not involve endangered or protected species.

### Data sampling

**Fig 1:**
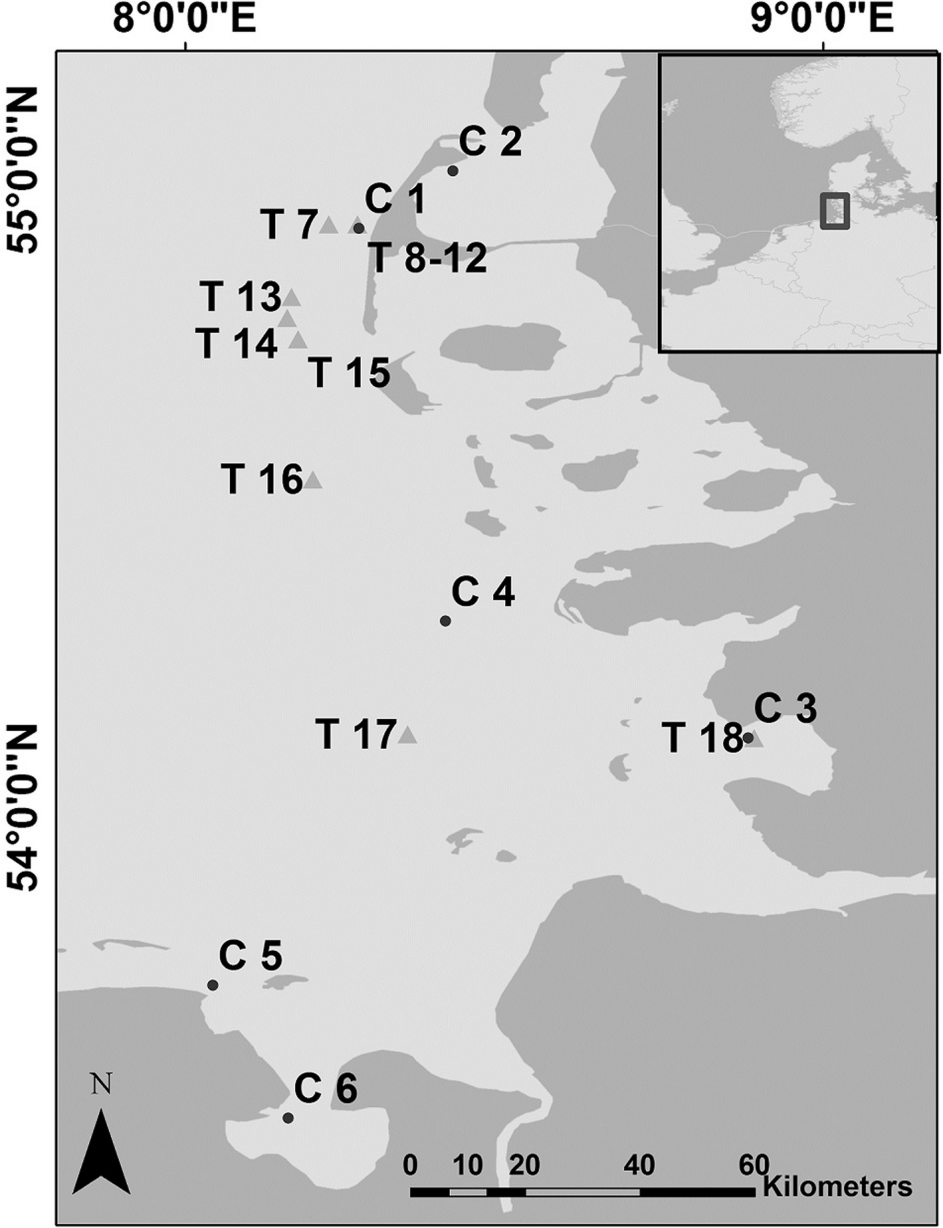
POD locations. T-POD stations (marked with a T and a triangle) were used in 2003–2006. C-POD stations (marked with a C and a circle) have been used since 2011 and are still running.

**Fig 2:**
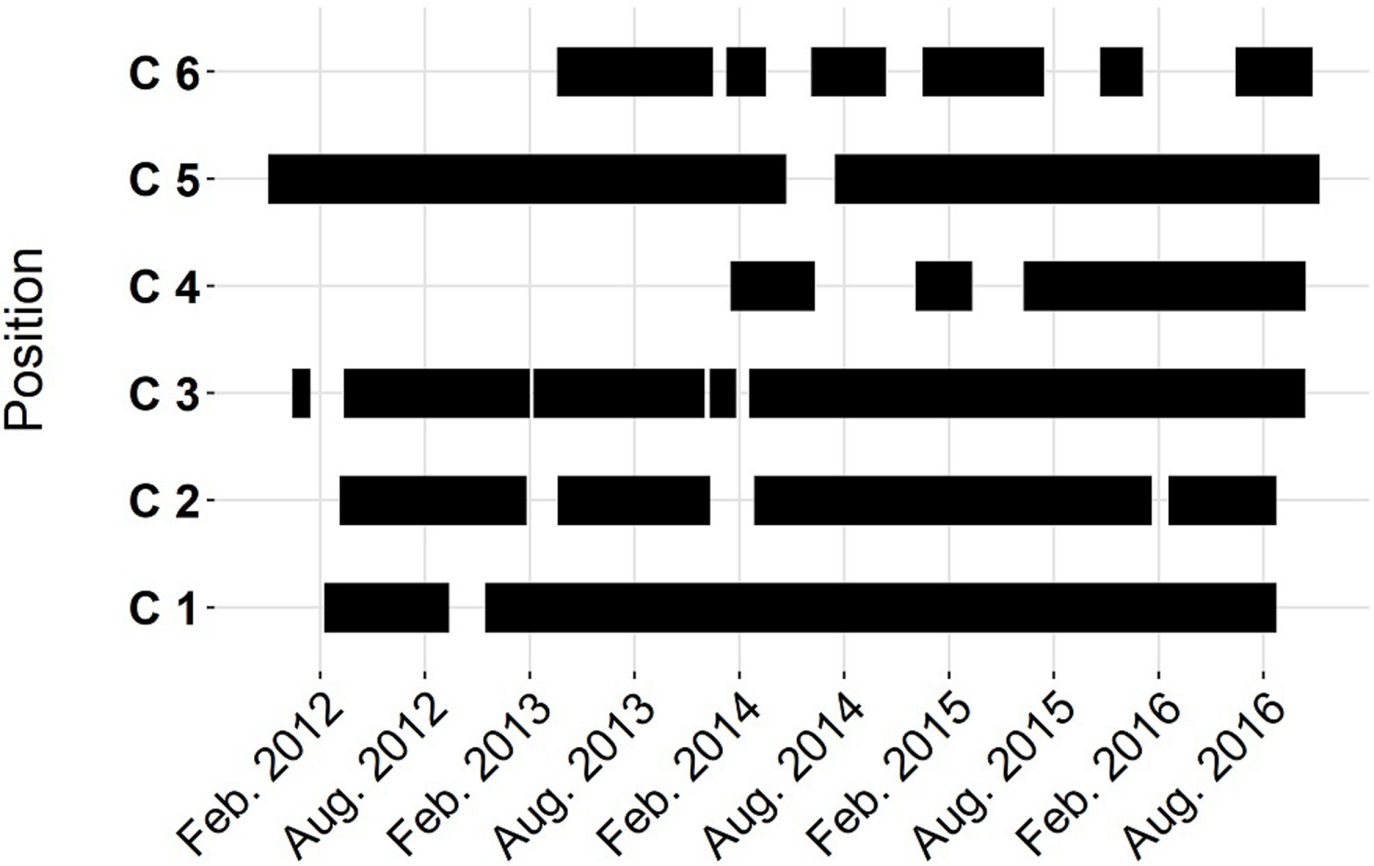
Timeline. Time scale for the recordings of C PODs respectively.

**Fig 3:**
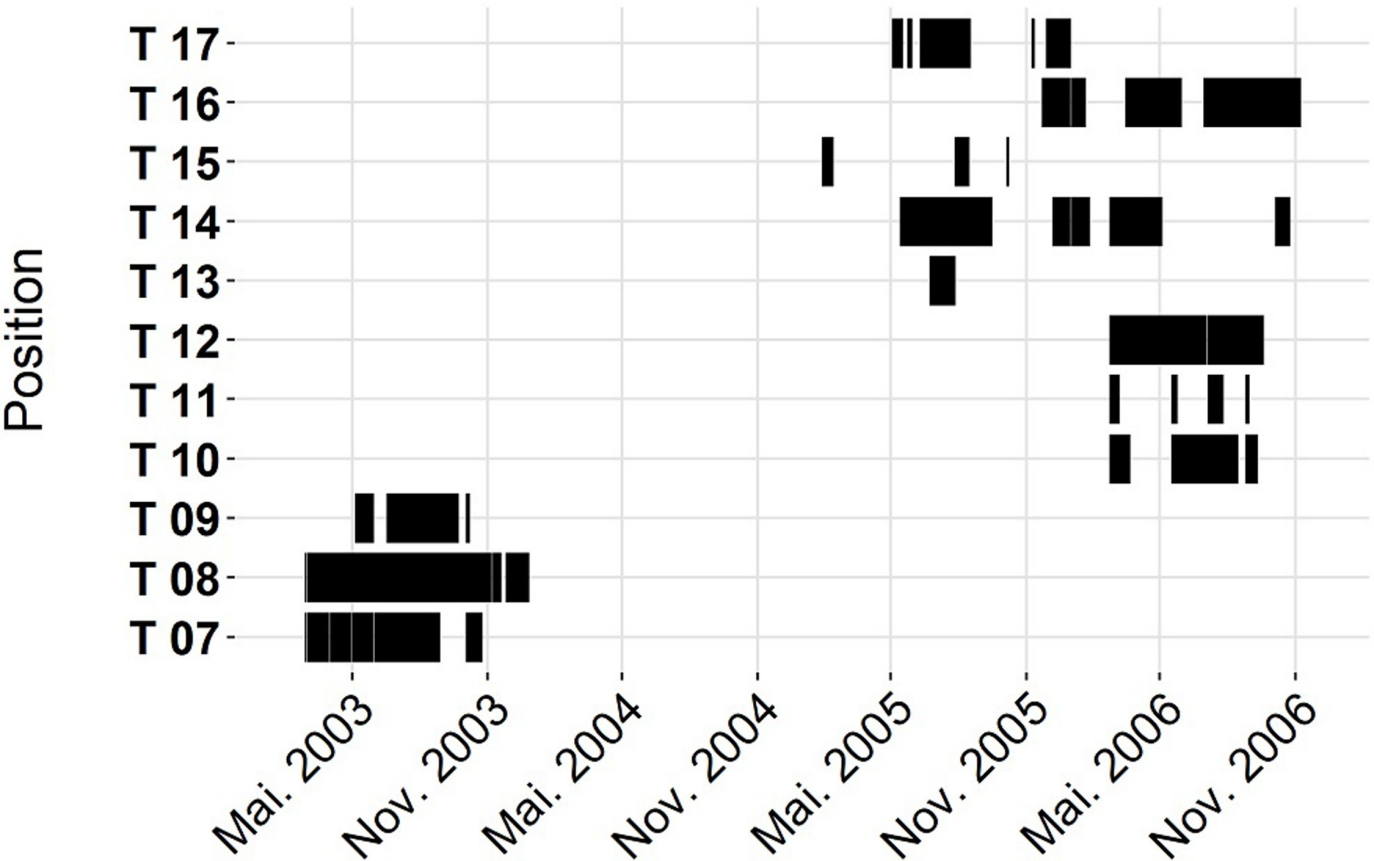
Timeline. Time scale for the recordings of T PODs.

Data were recorded by static acoustic monitoring in the German Wadden Sea area at 17 locations (Fig 1, permission from Federal Waterways and Shipping Administration (WSV) and Agency for Coastal Defence, National Park and Marine Conservation Schleswig- Holstein and Lower Saxony) with autonomous acoustic data loggers (T-PODs and C-PODs; www.chelonia.co.uk). Stations had a minimum distance from at least 500 m from the next one and covered the coast line and important estuary areas (Fig 1). The recording effort from C-PODs and T-PODs are given in Figs 2 and 3, respectively.

T- and C-PODs were moored two to five metres above the seafloor. They had one to two marker buoys and were connected with an 18-mm nylon rope to concrete anchors. Lifting bodies were used to maintain the units in an approximately vertical position, even under strong tidal currents. The PODs were set to only record when the devices were in a vertical position directed upwards and switch off when the inclination angel was greater than 82 degrees. Every two to three months the PODs were exchanged and ropes and equipment inspected. The PODs were regularly calibrated every few months [62].

There are different underlying detection mechanisms for T- and C-PODs. T-PODs compare the sound energy of two frequency bands of 90 and 130 kHz. If a certain ratio is reached they record the time point and duration of the event [63]. In comparison, C-PODs record tonal sounds and log the time, centre frequency, sound pressure level, duration and bandwidth of each click (www.chelonia.co.uk /cpod_downloads.htm). The detection rate of T-and C-PODs at the same position was compared in the wild for over one year of recordings [39]. The C-PODs recorded about twice as many detection positive minutes per hour as T-PODs with approximately linear relationship. This might influence the number of recorded events but as POD types and positions were analysed completely separately, no differences in effect size should affect the model outputs. For both devices, we applied a limit of logging 4096 clicks per minute and logging was stopped for the rest of the minute when the threshold was reached. This limit was set to save battery and memory capacity in noisy areas. The data were regularly checked for the amount of potential data loss. The median of minutes a day with 4096 clicks was mostly just above 0 percent and generally shows low background noise.

### Data analysis

Data were processed using the programs T-POD 8.19 and C-POD 2.044 (Chelonia Ltd). Built-in filtering algorithms (KERNO for C-POD and train classifier for T-POD) were used to select only clicks of harbour porpoises with a high likelihood (cetacean all and high/- moderate porpoise click trains respectively) of coming from a porpoise. All further analyses were carried out with the program R [64]. Inter-click-intervals (ICI) were calculated and data were scanned for buzzes which were defined as five consecutive clicks with ICI below ten milliseconds [47,59,65]. Data were grouped into click and buzz positive ten minutes (DP10MIN, BP10MIN) whereas BP10MIN was analysed from times when the porpoise was present (DP10MIN) only. Both were binary coded and not representing the specific number of detected clicks. DP10MIN and BP10MIN were then modelled dependent on abiotic variables (day time, tidal phase, day of year, year) that serve as a proxy for factors more meaningful for the animal (e.g. temperature, salinity, light availability).”

### Environmental data

Tidal data were derived from the X-Tide software at the POD positions [66]. Sun rise and sun set data were taken for one central position at 54.5N and 7.5E and calculated using the package “Maptools” in R. Time sequences of ebb and flood tide change constantly over time and time of sun rise and sun set changes over the season. To standardize tides and daylight radian values were calculated. The values 0 and 2 Pi describe the phase of tide and day cycles and represent high water and dawn respectively, while and Pi represents low water and dusk.

### Statistical analysis

Porpoise detections (expressed as the binary response variable DP10MIN, i.e. the probability of detecting at least one click train in a ten minute bin) and buzz occurrences relative to detections (expressed as the binary response variable BP10MIN, i.e. each the probability of detecting at least one buzz in DP10MIN) were analysed using time of day, tidal phase (referred to as tidal time) and the day of year (ordinal date numbers) as predictors. The buzz occurrence models (with the response variable BP10MIN) aim at estimating the likelihood that a porpoise present at the POD-position is feeding. Therefore 10-minute intervals in which no porpoise clicks were detected were excluded from this analysis. At each position analyses were conducted only when the recorded data covered more than 3 months. Separate models were calculated for each mooring position. This also meant that T-POD and C-POD data were not analysed within the same model. In order to correct for differences in harbour porpoise occurrence between years, year was included as a factor in all models that were based on multi-year records. We first calculated binary Generalized Additive Models (GAM). The cyclic predictors time of day, tidal time and day of year were modelled as cyclic cubic regression splines. In cases of stations where data was only available for part of the year, day of year was modelled as an ordinary cubic regression spline. Autocorrelation function (ACF) plots showed significant temporal autocorrelation of the residuals. Generalized Estimating Equations (GEE) can account for the observed temporal autocorrelation and have previously been used for the analysis of porpoise click detections [35,40]. Our general modelling approach followed their recommendations on incorporating splines in GEEs obtaining so-called GEE-GAMs. For specifying the correlation structure in the GEE-GAMs we grouped the data into blocks using a block size of 1000 minutes for T-PODs and 3000 minutes for C-PODs. These limits were selected based on a visual analysis of the ACF plots. We report full models for all mooring positions, i.e. all models include all predictor variables (i.e. day time, tidal phase, day of year and–if applicable–the factor year, which was included in all models for stations with multi-year recordings) and share the same internal covariate structure. No variable selection was performed, because all covariates were highly significant in the great majority of the models calculated for the different mooring positions. In such a situation, where all covariates are expected to have an effect on the response, it is generally desirable to control for the effect of a covariate even if it is not significant in a particular model [67]. Significance of the covariates was assessed using Wald’s tests. The models’ goodness of fit was evaluated based on the Area Under Curve (AUC) of the Receiver Operating Characteristic (ROC) and on presence-absence confusion matrices. Confusion matrices are two-way contingency tables that allow evaluation of model performance by tabulating the percentage of predicted presences and absences for both observed presences (in the first column) and observed absences (in the second column). For calculating the confusion matrices, the threshold for classifying a prediction as a presence was determined as the point at which the distance between the ROC curve and the 45° diagonal was maximal. ROC-AUC values can vary between zero and one with 0.5 representing the performance of a random predictor and one representing perfect prediction performance.

Additionally, separate models of buzzing behaviour (BP10MIN as dependent) were calculated for each of the four calendric seasons as replacement for day of year, to investigate whether generalizable patterns for the seasons can be found.

## Results

At all POD stations echolocation clicks of harbour porpoises were regularly recorded and recording effort was 9888 days from 17 different positions. At four POD sites T11, T13, T15 and T18 recorded data was less than 3 months and they were therefore excluded from further analyses. Results of the GEE-GAM models indicate highly significant effects of day of the year, tide and time of the day on harbour porpoise detections at most POD sites (Table 1).

**Table 1:** Summary of model results.

**DP10MIN**														
Model			confusion matrix			Wald´s Test								
POD Position/type	Recording effort [d]			Observed		Day of year		Daytime		Tidal cycle		year		
		AUC	Prediction	Porpoise	No porpoise	X²	p values	X²	p values	X²	p values	DF	X²	p values
C 1	1593	0.62	Porpoise	44%	24%	82	< 0.001	9	0.094	24	< 0.001	4	0	0.979
			No porpoise	56%	76%									
C 2	1470	0.65	Porpoise	63%	41%	18	0.006	6	0.263	33	< 0.001	4	35	< 0.001
			No porpoise	37%	59%									
C 3	1677	0.69	Porpoise	56%	30%	87	< 0.001	42	< 0.001	56	< 0.001	5	87	< 0.001
			No porpoise	44%	70%									
C 4	735	0.66	Porpoise	64%	42%	28	< 0.001	17	0.005	16	0.002	2	62	< 0.001
			No porpoise	36%	58%									
C 5	1749	0.63	Porpoise	70%	52%	24	< 0.001	15	0.009	67	< 0.001	5	19	0.002
			No porpoise	30%	48%									
C 6	890	0.76	Porpoise	64%	25%	115	< 0.001	18	0.003	156	< 0.001	3	20	< 0.001
			No porpoise	36%	75%									
T 7	206	0.79	Porpoise	80%	34%	113	< 0.001	27	< 0.001	13	0.014			
			No porpoise	20%	66%									
T 8	298	0.6	Porpoise	72%	58%	9	0.186	7	0.248	14	0.006			
			No porpoise	28%	42%									
T 9	131	0.65	Porpoise	46%	24%	38	< 0.001	18	0.003	2	0.719			
			No porpoise	54%	76%									
T 10	136	0.66	Porpoise	68%	43%	156	< 0.001	7	0.199	16	0.003			
			No porpoise	32%	57%									
T 12	209	0.63	Porpoise	72%	52%	93	< 0.001	9	0.11	14	0.007			
			No porpoise	28%	48%									
T 14	269	0.65	Porpoise	65%	43%	44	< 0.001	19	0.002	39	< 0.001			
			No porpoise	35%	57%									
T 16	269	0.63	Porpoise	60%	42%	37	< 0.001	20	0.001	15	0.004			
			No porpoise	40%	58%									
T 17	130	0.7	Porpoise	63%	35%	75	< 0.001	15	0.011	51	< 0.001			
			No porpoise	37%	65%									
**BP10MIN**														
Model			confusion matrix			Wald´s Test								
POD position/type	recording effort[d]			Observed		Day of year		Daytime		Tidal cycle		year		
		AUC	Prediction	Buzzing	No buzzing	X²	p values	X²	p values	X²	p values	DF	X²	p values
C 1	1593	0.58	Buzzing	59%	48%	32	< 0.001	32	< 0.001	68	< 0.001	4	2	0.677
			No buzzing	41%	52%									
C 2	1470	0.58	Buzzing	74%	62%	622	< 0.001	14	0.016	24	< 0.001	4	122	< 0.001
			No buzzing	26%	38%									
C 3	1677	0.6	Buzzing	57%	43%	42	< 0.001	7	0.187	28	< 0.001	5	68	< 0.001
			No buzzing	43%	57%									
C 4	735	0.61	Buzzing	66%	49%	191	< 0.001	29	< 0.001	14	0.008	2	0	0.924
			No buzzing	34%	51%									
C 5	1749	0.57	Buzzing	55%	44%	52	< 0.001	27	< 0.001	25	< 0.001	5	251	< 0.001
			No buzzing	45%	56%									
C 6	890	0.63	Buzzing	58%	39%	58	< 0.001	25	< 0.001	101	< 0.001	3	68	< 0.001
			No buzzing	42%	61%									
T 8	298	0.64	Buzzing	69%	48%	107	< 0.001	280	< 0.001	70	< 0.001			
			No buzzing	31%	52%									
T 14	269	0.63	Buzzing	60%	41%	159	< 0.001	25	< 0.001	14	0.006			
			No buzzing	40%	59%									
T 16	269	0.57	Buzzing	62%	51%	44	< 0.001	12	0.04	11	0.022			
			No buzzing	38%	49%									

Summary of the model results: AUC and confusion matrix for predicted and observed porpoise detection (DP10MIN) and for predicted and observed buzzes (BP10MIN). Wald´s test results of the GEE-GAM model for DP10MIN and BP10MIN of the different stations. The covariates day of year (DF = 6), daytime (DF = 5) and tidal cycle (DF = 4) are presented.

**Fig 4:**
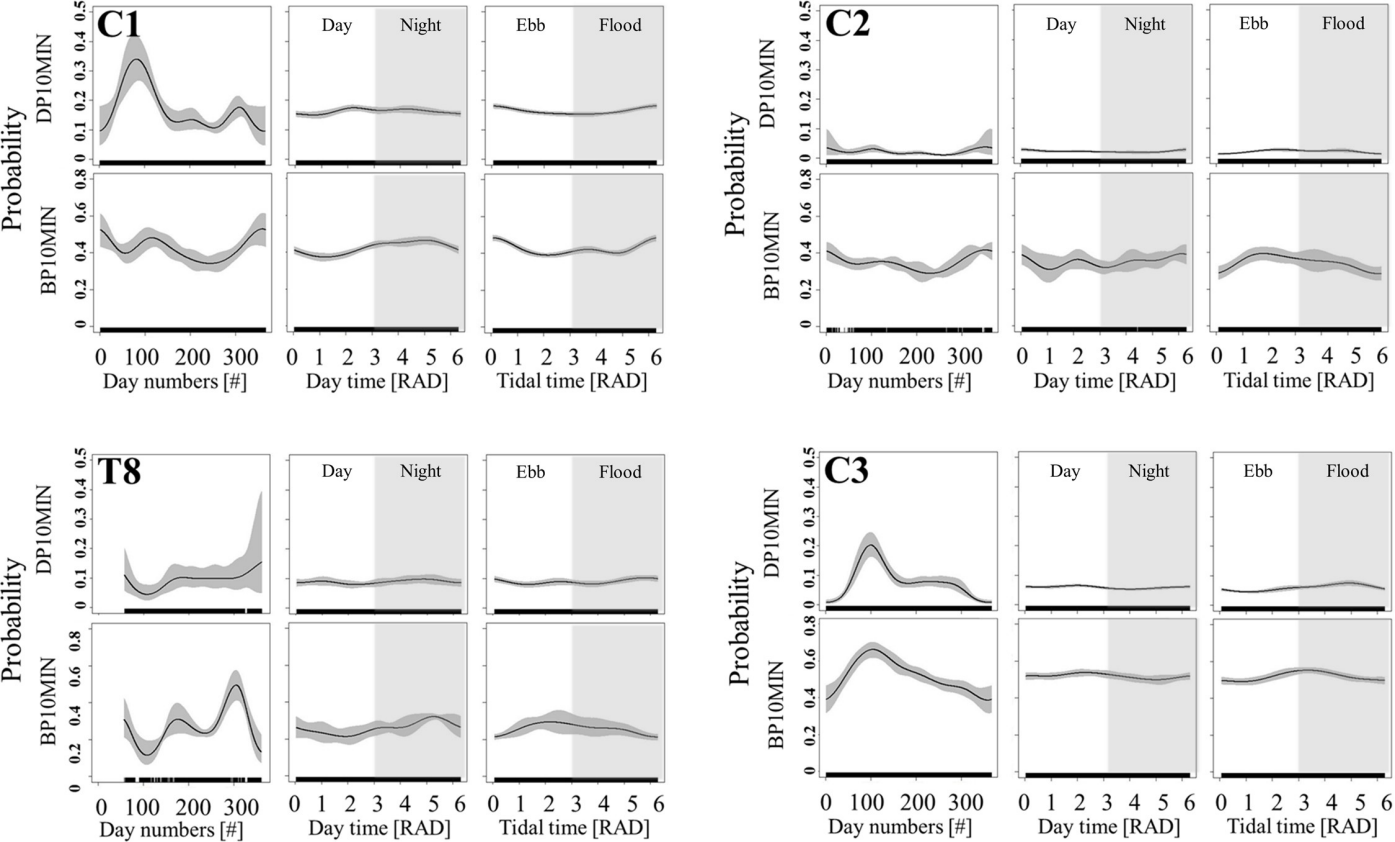
Model results. Effects of season, daytime and tide on the probability that either a click is detected (DP10MIN) or a buzz (BP10MIN) is observed in a 10-minute interval. Estimates of the GEE-GAM model are plotted for four selected sites (C1, C2, T8 and C3). Grey areas represent associated 95% confidence intervals. The values for day time of 0 and 6 are representing dawn and the transition between the white and grey background is equal to dusk. Day-time is with a white background and night-time is shaded in grey. Similarly, for tide 0 and 6 are representing high water and the transition between white and grey areas are equal to low water. Ebb tide is with a white background and flood tide is shaded in grey. (For all positions see supplement).

At most sites, the predictor variable with highest chi-squared value and thus highest explanatory power is day of year for porpoise detections (Table 1). A peak in detections can be found around day 100 (March to May) for 5 stations while all others showed a highly variable seasonal variation. Several years of observations exist only for the C-POD stations and effects of year could be shown at most POD locations. On a smaller scale, we found that effects of tide and daytime on porpoise detections were highly variable and site specific. At most sites detections were slightly elevated at high tide but also peaks of detections at low tide and at flood tide were found (Table 1 and Figs 4 and S3 and S6). Diel patterns were found for most of the POD stations, with detection peaks at night, day or twilight (Table 1 and Figs 4 and S2 and S5).

Visual comparison of the model outputs for porpoise detections and buzzing behaviour show variable effects. Over days of the year, at some positions an increase in buzzes was detected even when detections of porpoises were going down (C1, T8, C4, C6). On the opposite, detection of buzzes also increased with increasing porpoise detections (C2, C3, C5, T14, T16; Figs 4 and S1 and S4).

BP10MIN were significantly influenced by most factors but very complex patterns were found across the different stations (Table 1). When visually grouping the station as closer to the coast (C1, C2, C3, C5, C6, T8, T9, T10, T12, Fig 1) and further away (C4, T7, T14, T16, T17, Fig 1) similar pattern for day of the year (C1, C3, C5, C6 and T14, T16, Figs 4 and S4) and tide (C2, C3, C5, T8, Figs 4 and S5) were found. A clear trend for detections are in-between high and low tides when current speed was likely to be highest (C2, C4, C6, T12, T14, T16, Fig 4). However, high probabilities for buzzes at high tide were also detected at two sites (C1, T7). A very site-specific pattern of diel behaviour could be seen for the different stations (Fig 4) showing nocturnal, diurnal and dusk/dawn pattern.

At the neighbouring POD positions C1 and C2, tide showed opposite effects on DP10MIN and BP10MIN (Fig 4). These two PODs were in close proximity to each other but were differently affected by the tides.

The factor year was significant at most stations for DP10MIN and BP10MIN indicating different patterns over the years. Only at one station for porpoise detections and at two stations for buzzing porpoises the effect of the year was not significant indicating stable pattern over the years of POD deployment.

**Fig 5:**
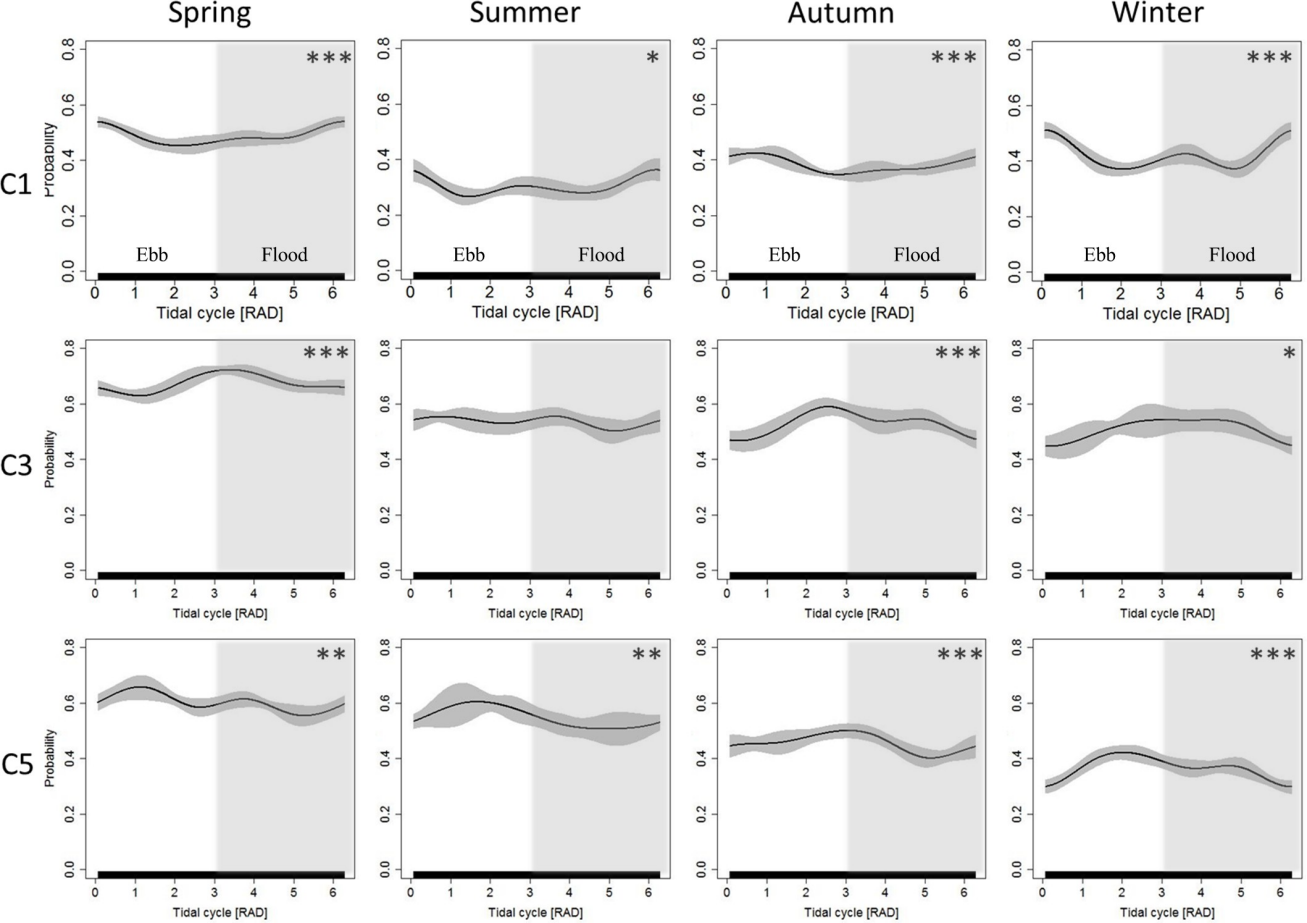
Seasonal tidal effects on detected buzzes. Effect of tidal time on the probability that a buzz is observed in a 10-minute interval (BP10MIN). Estimates of GEE-GAM models for each calendric season and POD position (C1, C3, C5) are shown. Grey areas represent associated 95% confidence intervals. The values for tide 0 and 6 are representing high water and the transition between white and grey areas are equal to low water. Ebb tide is with a white background and flood tide is shaded in grey. Model significance is symbolled with asterisk (* = p ≤ 0.05, ** = p ≤ 0.01, *** = p ≤ 0.001).

**Fig 6:**
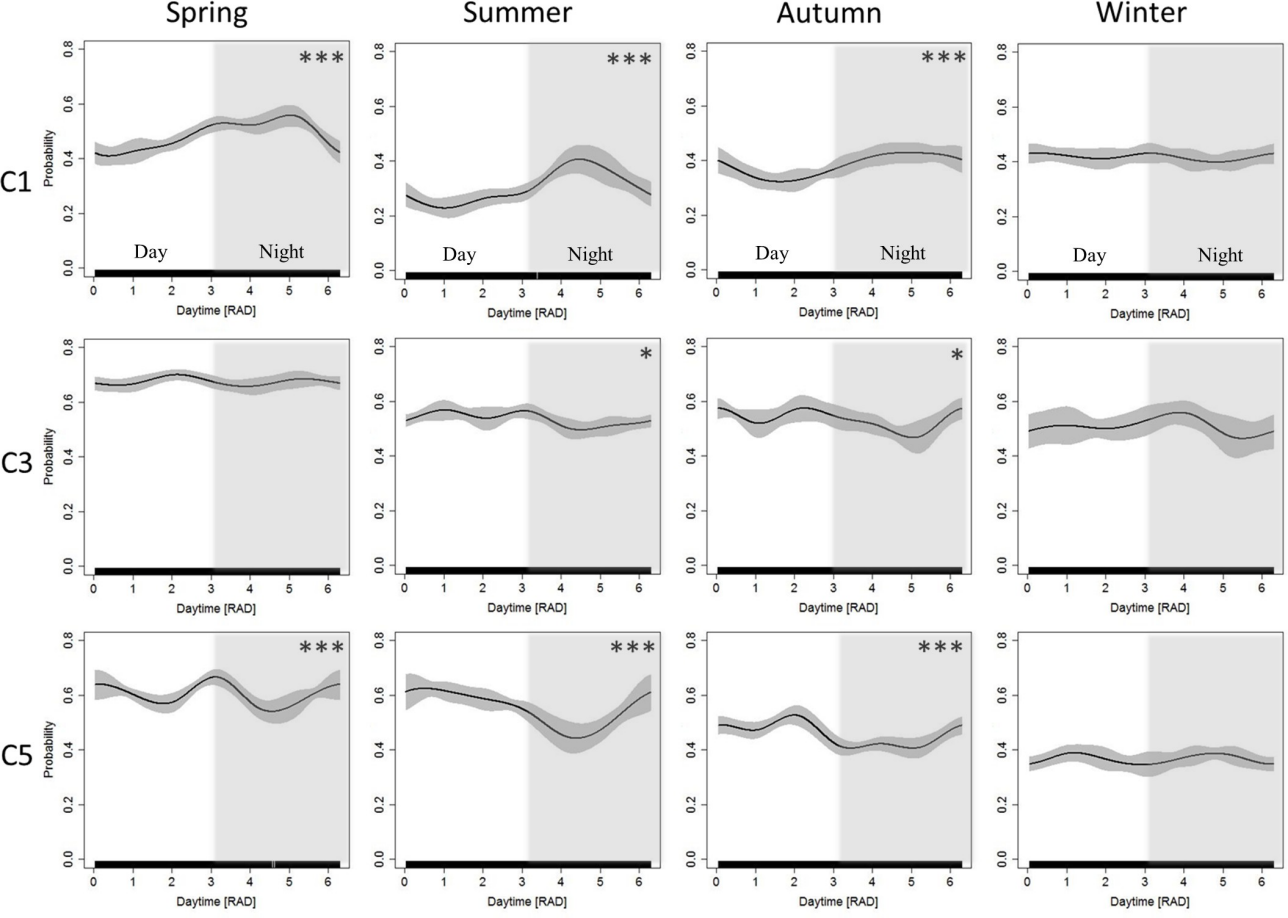
Seasonal daytime effects on detected buzzes. Effect of the time of day on the probability that a buzz is observed in a 10-minute interval (BP10MIN). Estimates of GEE-GAM models for each calendric season and POD position (C1, C3, C5) are shown. Grey areas represent associated 95% confidence intervals. The values for day time of 0 and 6 are representing dawn and the transition between the white and grey background is equal to dusk. Day-time is with a white background and night-time is shaded in grey. Model significance is symbolled with asterisk (* = p ≤ 0.05, ** = p ≤ 0.01, *** = p ≤ 0.001).

The season-specific effects of tide and daytime on porpoise detection probability were analysed (Figs 5 and 6) for the 3 C-POD sites with the most complete coverage of all seasons (Table 1). At these stations the impact of the tide and time of the day changed over season. At position C1 there was a clear nocturnal effect from spring to autumn, which was not detected in winter. At this time of the year, however, the effect size of tide increased at the specified position.

The AUC-values in Table 1 are variable and range from 0.6 to 0.79 for DP10MIN and from 0.57 to 0.64 for BP10MIN. Generally, models calculated at stations with only significant factors have higher values in the AUC-values.

## Discussion

We analysed long term acoustic data of harbour porpoises in the German Wadden Sea and found strong and statistically significant seasonal, tidal and daylight effects on detections and buzzing behaviour at the different deployment positions over the time period from 2003 to 2016.

A high probability in detecting porpoises and buzzes in the Wadden Sea especially at POD positions close to the coast (Figs 4 and S1 and S4) was found in spring. This is in line with aerial observations and supports a seasonal on/offshore movement pattern of porpoises and a general movement of porpoises from other areas into the German North Sea in spring [14,16]. General seasonal movement [30] might reflect porpoise responses to different prey types and prey abundance [29]. Seasonal hot spots in prey abundance might additionally lead to a more aggregated porpoise distribution in spring and a more even distribution of porpoises in the North Sea in the rest of the year [6,29]. Our results show that the Wadden Sea might also be an important feeding and breeding habitat for porpoises with a peak in detected buzz positive intervals in spring. This is in accordance with previous analyses of stranded and bycaught porpoises in the German North Sea indicating a higher biomass of ingested prey in spring [29]. This time is just before the most common time of porpoises giving birth in June-July [68]. In this time period female cetaceans have to gain weight for the time of lactation [69], when they cannot gather enough prey to replenish their energy resources [70]. Shallow Wadden Sea areas have higher fluctuations in water temperature, creating areas of higher temperatures, compared to the neighbouring North Sea. This could be beneficial for animals due to decreased energy expenditure caused by maintenance of core temperature. Later in the year, we found a decrease in detections suggesting that most calves are not born in the Wadden Sea, or that some animals move elsewhere during that crucial time.

Nocturnal and diurnal pattern were found in porpoise detections and foraging behaviour in our data (Figs 4 and S2 and S5). These significant but very site-specific pattern could be directly linked to their prey and might be explained by differences in physical parameters, such as depth, slope and sea bed composition [45,71] of the POD sites. In the Baltic Sea, acoustic detections have been suggested to be linked to pelagic prey of porpoises, e.g. herring, that could be easier accessed at night [52]. Herring shows decreased predator avoidance mechanisms during the night, being closer to the surface in less aggregated schools and reduced swimming speed [72,73]. This could be reflected in porpoise behaviour as supported by a previous study [4] and therefore buzz rates may increase at certain times of the day due to porpoises following the vertical distribution of their prey. In contrast to these findings from the Baltic Sea a major prey item of harbour porpoises in the North Sea, the sandeel [29,74,75], shows a diurnal vertical migration pattern feeding on surface zooplankton during the day and buries itself in sand at night especially in spring time [76]. This would explain diurnal patterns in our data in addition to nocturnal patterns possibly linked to other prey species. Additionally, seasonal changes have diverse impacts on different prey fish types, and therefore might alter the distribution of the opportunistic feeding harbour porpoises in different habitats.

An alternative explanation to an increased echolocation at night could be to compensate the loss of visual information due to darkness [47]–a large advantage for nocturnal predators. A similar nocturnal increase in echolocation detection should then be expected in all odontocetes but that has not been found in studies of bottlenose dolphins [26] and Heaviside dolphins [77]. This was proved in captivity were echolocation activity did not increase when a porpoise was blindfolded [56] and may strengthen the hypothesis that different nocturnal and diurnal patterns are linked to the distribution of their prey in dependency of the habitats.

In coastal zones, inflows from rivers, estuaries and tidal flats increase the concentration of organic matter [10]. Therefore, strong tidal currents or different tidal stages could influence prey availability and porpoise abundance indirectly. Different water movement could more directly physically impact the distribution of their prey as the prey fish might be concentrated in areas with high differences in current speed. In the current study, we found a strong impact of tides on porpoise detection rates and detection of buzzes. Porpoises were mostly detected at high tide but also at times of high current speeds, at flood tide and at low tide (Figs 4, 5 and S3 and S6 and Table 1). Buzzes were mostly detected in high current speed and at ebb tide (C2, C4, C5, C6, T8, T14, T16; Figs 4 and S6). Our findings are supported by a short-term study which found an increase in porpoise detections and encounter duration when an energetic tidal jet occurred and clearly showed an attraction for porpoises to strong currents [32]. For general conclusions on porpoise distribution more complex measurements than tidal factors alone are needed and local differences in topography and tides may have various effects at different sites [40].

Occurrences of buzzes of porpoises were analysed in all detection positive intervals and a visual comparison of the occurrence of detections and buzzes show that they are not automatically linked. At some stations (C1, T8, C4, C6), an increase in buzzes was detected even when detection of porpoises was going down at different times over the year. However, at other stations (C2, C3, C5, T14, T16; Figs 4 and S1 and S4), detection of buzzes were increasing with increased porpoise detections. This shows that the method of passive acoustic monitoring with T/C-PODs is sensitive enough to indicate different behaviours and can help detecting buzzes as proxy for feeding behaviour in a localised position.

Potential feeding events, buzzes, were analysed for different calendaric seasons and showed a site-specific diel and tidal pattern. When the diel effect was less pronounced (C1 winter, Fig 6) the effect size of tide increased (Fig 5). This might generally suggest a stronger effect of currents and when current spends are low day time effect might be more pronounced. However, strong seasonal differences can be seen. This highlights the importance of yearlong observations to draw general conclusions on buzzing and detections.

Inter-annual differences of detections and buzzes at most POD sites show a variable porpoise behaviour over the years. This supports the idea that porpoise behaviour in the Wadden Sea is much more diverse than previously known. Generally, different patterns found in the long-term data set tend to be highly site specific. For example, even at neighbouring POD positions C1 and C2 an opposite effect of the tide on porpoise detection and buzzes can be found. These positions, even though they are in close proximity are differently affected by tides. C1 is right at the open coast of the island Sylt whereas C2 is sheltered behind the island in much calmer water. At the more exposed position C1 porpoises and buzzes were detected significantly more at high water, whereas an increase at low water was found at the more sheltered position C2. This may indicate that environmental factors with cyclic variation may lead to regular concentrations of porpoises or it could indicate that site fidelity of harbour porpoises is stronger than previously assumed. Site fidelity, in broad terms, is the inclination of an animal to remain or revisit an area formerly occupied [78]. In theory it optimizes the energy expenditure of an animal in a steady or fast changing environment, depending on prey availability and other biologically significant factors. Reports from a photo-identification study from Washington State (USA) suggest that at least some porpoises show high site fidelity [79] and this has also been hypothesized for porpoises from Morro Bay, California [80]. However, telemetry studies from the Danish Belt Sea and Skagerrak waters aimed at estimating population home ranges [81]. They found key habitats potentially associated with higher dispersal but for a completely different population and habitat. Since no porpoise was refitted with a logger after a year, when a seasonal migration would start over again the perspective on longer time scales is limited. Photo-ID or long-term telemetry studies focussing on the Wadden Sea are necessary to validate the hypothesis of strong site fidelity which currently cannot be substantiated using static acoustic monitoring due to the inability to identify individuals.

Distinct patterns in acoustic communications calls corresponding to 5 differing behavioural classes have been identified in captive animals [82]. Four of these call types contain high click rates with ICIs below 10 ms, accompanied by a sudden drop in ICI. Free ranging harbour porpoises were shown to produce communication calls in 7.7 to 31.5% and receive communication calls from conspecifics in 1.7–3.6% (4 single animals) and 18.5–34.4% (2 mother calf pairs) of observation minutes, indicating that social interactions are of higher importance than previously assumed [83]. Foraging buzzes were furthermore shown to be clearly distinct from communication calls [83]. Unfortunately, the discrimination of communication and foraging buzzes [83] cannot be conducted in comparable manner for PODs since movement data and echoes of prey cannot be determined. Nevertheless, foraging buzzes are produced more frequently (17.8–75.8%, on average 52.6% of minutes) [84] than communication buzzes and harbour porpoise group sizes are typically small in the North Sea (average group size of 1.24 animals) [30]. Therefore, buzzes will represent the majority of the classified sequences within the here presented study and we suggest that increases in buzz occurrence should mostly reflect increases in individual feeding rate. The frequent presence of communication sequences and responding conspecifics could furthermore also be an indirect indicator of foraging, possibly representing cooperative foraging strategies [83]. Due to the described limitations with data logged by static acoustic recorders and additionally having data with incomplete click sequences, the sudden decrease of ICI below 10 ms is to date the best indicator for this method to classify foraging and has been regularly applied [26,47,49,52,85,86,87].

While using static acoustic monitoring throughout our study, we have differences in the acquisition of data. The biggest contrast between the different POD types is due to the different deployment times (Figs 2 and 3). At all C-POD positions data acquisition was over several years whereas the T-POD data was less than 300 days with a minimum recording time of 130 days. Therefore, the model output of T-POD data is restricted to the recording times of the PODs and rather reflect seasonal than general patterns especially for shorter recording times. For both POD types, the evaluation of the goodness of fit for the calculated models was done with AUC of the ROC. Our AUC-values are ranging between 0.6 to 0.79 for DP10MIN and from 0.57 to 0.64 for BP10MIN. This indicates that our predictor variables can only partly explain porpoise detections and that occurrence of buzzes are explained with less certainty than detections. Other factors that were not recorded might be influencing the behaviour of the porpoises as well. Compared to a study using a similar method for analysing porpoise detections [35] our study has similar or slightly lower AUC-values. In Benjamins study the AUC-values for porpoise detections ranged from 0.59 to 0.78 at one site and between 0.64 and 0.88 at the other site. Their recording effort was 58 and 55 days and therefore much shorter than in our study. The fact that in much shorter recording efforts AUC values are slightly higher might be another indicator that tidal or day time pattern might be relatively stable over some months [35] but more variable over years (our study).

Another difference between the two kinds of PODs are the different deployment depths which could lead to different detection rates as these are influenced by the distance of the POD to the sea floor [88]. Due to a general variation in dive depth of porpoises during pelagic feeding we assume that deployment depth in the tidal waters of the German North Sea is of lesser importance. However, the setup of the POD will mostly detect pelagic feeding and is likely to miss bottom grubbing [52].

Concluding, our results show that the Wadden Sea is an important habitat for harbour porpoises. It is used for feeding and especially at times before and in the breeding season. Porpoises use the Wadden Sea throughout the year but at different intensities at the different POD positions. Additionally, we found effects of time of the day and of different influences of the tides on detections and buzzing behaviour of porpoises. However, very site-specific results with annual differences of detections and buzzes indicate a high flexibility of porpoises in a highly variable environment. Factors like day time and tide were shown to vary in their influence on porpoises in different season at the same positions. Our results based on a multi-year dataset thus indicates that studies drawing conclusions on porpoise behaviour based on short-term datasets might rather reflect seasonal than general patterns and should be interpreted accordingly.

Our study gives new insights into harbour porpoise occurrence and buzzing behaviour in the German Wadden Sea and it also raises interesting research questions like how porpoises use specific situations efficiently for feeding, how they predict prey occurrence and if individuals visit the same sites in subsequent years.

## Supporting information

S1 Fig Daily variance of porpoise detection. All GEE-GAM results for DP10MIN probability at each POD position, shown are thin plate regression splines showing daily variance over year.
(PDF)

S2 Fig Daytime dependency of porpoise detection. All GEE-GAM results for DP10MIN probability as a function of time of the day at each POD position,the rad values of 0 and 2 Pi are representing dawn and Pi is equal to dusk.
(PDF)

S3 Fig Tidal dependency of porpoise detection. All GEE-GAM results for DP10MIN probability in dependency of tide at each POD position separate, the rad values for tide of 0/2 Pi are representing high water and Pi is equal to low water.
(PDF)

S4 Fig Daily variance of porpoise buzzes. All GEE-GAM results for BP10MIN probability at each POD position, thin plate regression splines show daily variance over year.
(PDF)

S5 Fig Daytime dependency of porpoise buzzes. All GEE-GAM results for BP10MIN probability in relation to time of the day at each POD position, the rad values of 0/2 Pi are representing dawn and Pi is equal to dusk.
(PDF)

S6 Fig Tidal dependency of porpoise buzzes. All GEE-GAM results for BP10MIN probability in relation to tide at each POD position separate, the rad values for tide of 0/2 Pi are representing high water and Pi is equal to low water.
(PDF)
