# Supplementary material for: Time and tide: Seasonal, diel and tidal rhythms in Wadden Sea Harbour porpoises (Phocoena phocoena)
Source: PLoS One. 2019 Mar 20;14(3):e0213348. doi: 10.1371/journal.pone.0213348 (PMC6426179; doi:10.1371/journal.pone.0213348)
Supplement: S2 Fig — All GEE-GAM results for DP10MIN probability as a function of time of the day at each POD position,the rad values of 0 and 2 Pi are representing dawn and Pi is equal to dusk. (PDF) [file pone.0213348.s002.pdf]

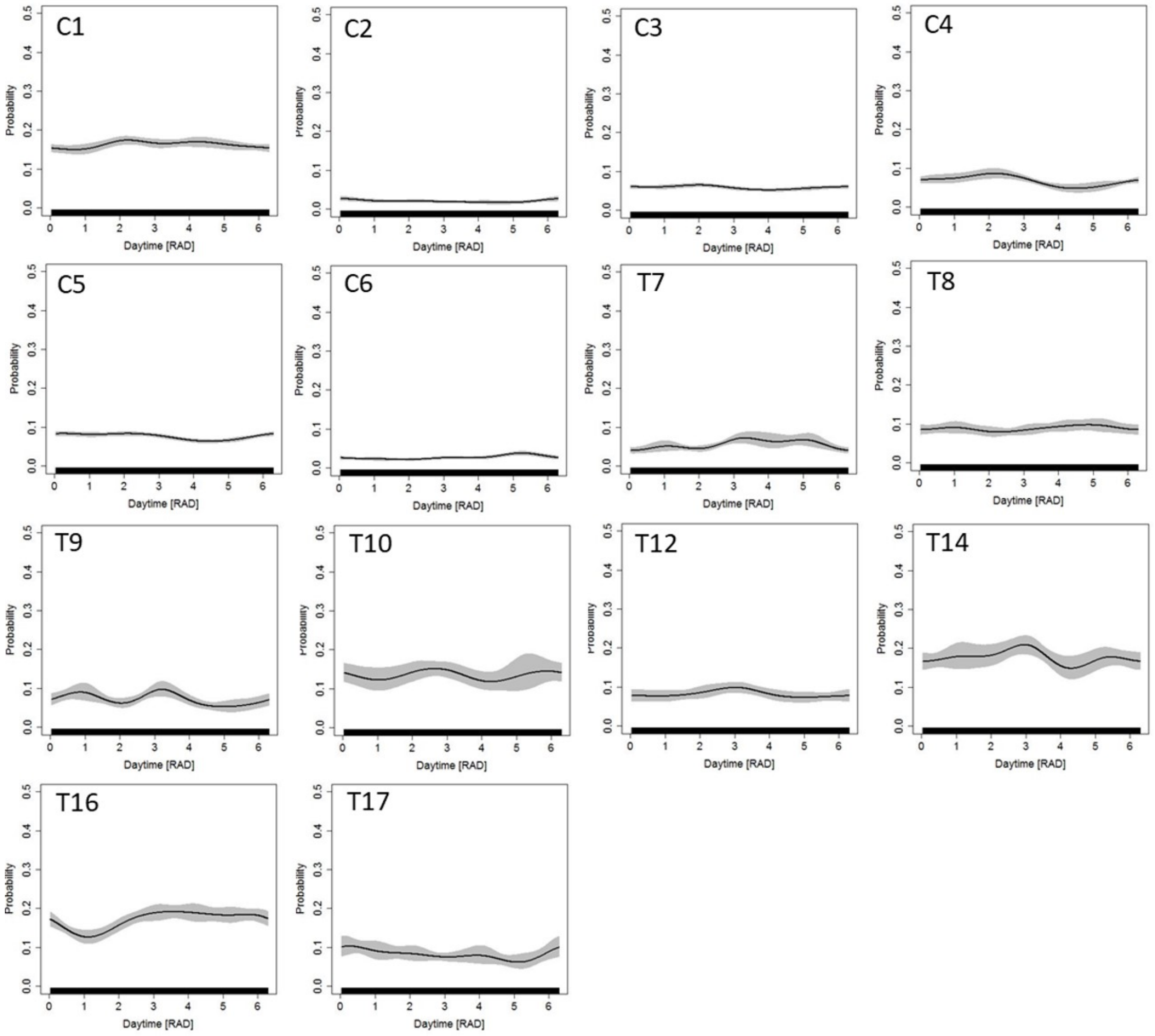

S2 Fig. Daytime dependency of porpoise detection. All GEE-GAM results for DP10MIN probability as a function of time of the day at each POD position,the rad values of 0 and 2 Pi are representing dawn and Pi is equal to dusk.
