# Supplementary material for: Time and tide: Seasonal, diel and tidal rhythms in Wadden Sea Harbour porpoises (Phocoena phocoena)
Source: PLoS One. 2019 Mar 20;14(3):e0213348. doi: 10.1371/journal.pone.0213348 (PMC6426179; doi:10.1371/journal.pone.0213348)
Supplement: S4 Fig — All GEE-GAM results for BP10MIN probability at each POD position, thin plate regression splines show daily variance over year. (PDF) [file pone.0213348.s004.pdf]

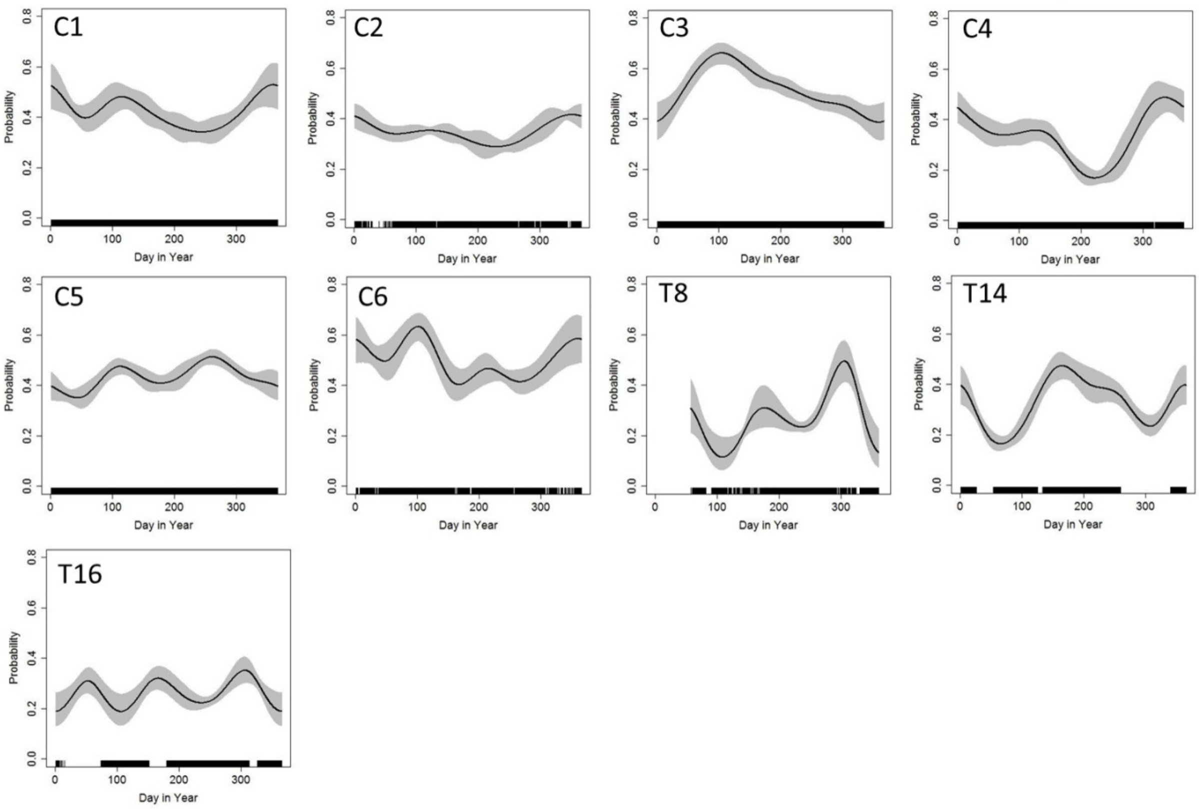

S4 Fig. Daily variance of porpoise buzzes. All GEE-GAM results for BP10MIN probability at each POD position, thin plate regression splines show daily variance over year.
