# Supplementary material for: Time and tide: Seasonal, diel and tidal rhythms in Wadden Sea Harbour porpoises (Phocoena phocoena)
Source: PLoS One. 2019 Mar 20;14(3):e0213348. doi: 10.1371/journal.pone.0213348 (PMC6426179; doi:10.1371/journal.pone.0213348)
Supplement: S6 Fig — All GEE-GAM results for BP10MIN probability in relation to tide at each POD position separate, the rad values for tide of 0/2 Pi are representing high water and Pi is equal to low water. (PDF) [file pone.0213348.s006.pdf]

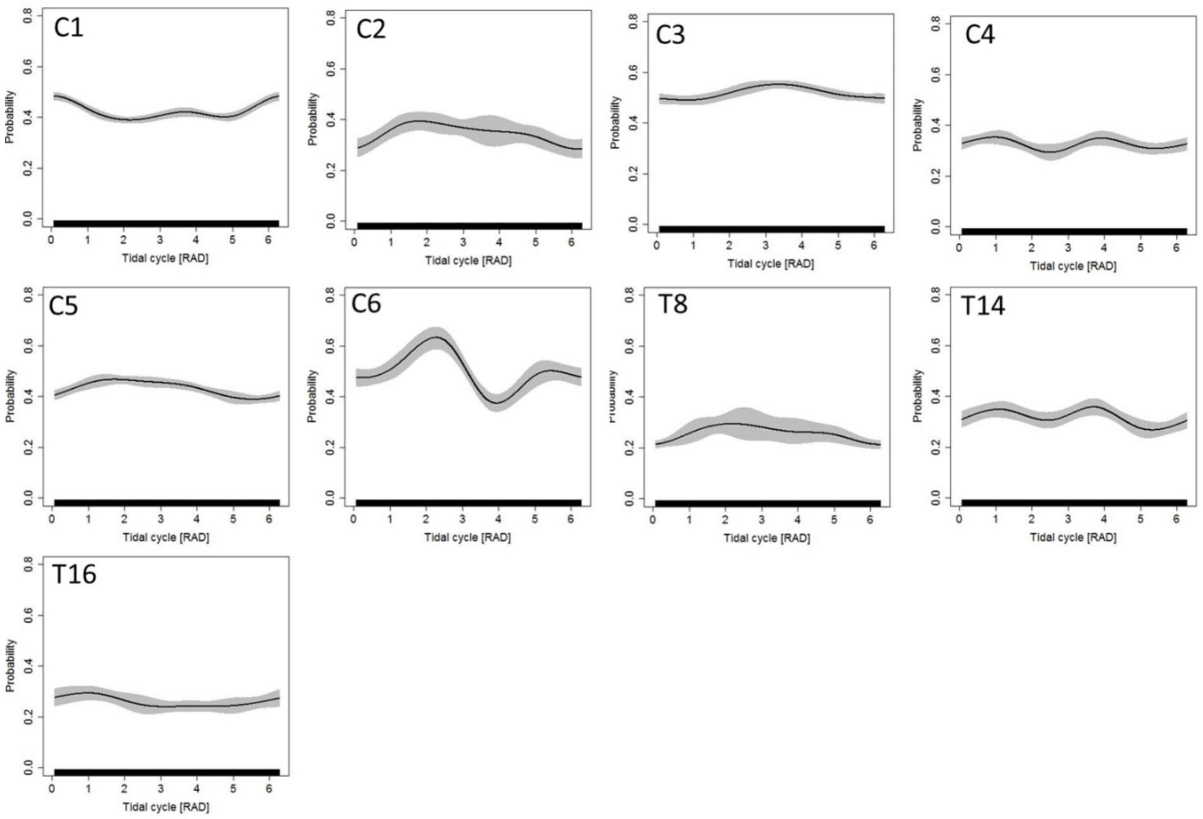

S6 Fig. Tidal dependency of porpoise buzzes. All GEE-GAM results for BP10MIN probability in relation to tide at each POD position separate, the rad values for tide of 0/2 Pi are representing high water and Pi is equal to low water.
